# Finesse in Forehead Lifting—Asymmetric Ligament Release for Asymmetric Brow Ptosis

**DOI:** 10.1007/s00266-026-05718-1

**Published:** 2026-03-19

**Authors:** Henry Bair, Tiffany S. Cheng, Sathyadeepak Ramesh

**Affiliations:** 1 Oculoplastic and Orbital Surgery Service, Wills Eye Hospital, Sidney Kimmel Medical College of Thomas Jefferson University, Philadelphia, PA USA; 2 The Center for Eye and Facial Plastic Surgery, Somerset, NJ 08873 USA

**Keywords:** Brow lift, Endoscopic brow lift, Forehead lift, Deep plane forehead lift, Brow asymmetry, Brow ptosis

## Abstract

**Background:**

Asymmetric brow ptosis presents a unique surgical challenge. It has been traditionally managed with full deep-plane release of the forehead retaining ligaments, which often leads to overcorrection or persistent asymmetry. This case series evaluates an approach using titrated asymmetric ligament release and selective fixation to address brow asymmetry while minimizing complications.

**Methods:**

We conducted a retrospective analysis of 50 consecutive patients (average age: 62.1 years) with preoperative brow asymmetry greater than 2 mm who underwent endoscopic brow lift between 2022 and 2024. All patients underwent bilateral temporal dissection and fixation. The surgical refinements included extended lateral ligament release (lateral release to the helical root performed on 100% of ptotic sides), selective medial dissection (performed on 62% of ptotic sides), and unilateral paramedian fixation (performed on 25% of cases). We evaluated postoperative symmetry, defined as less than 2 mm asymmetry, and any complications using standardized photographs and clinical assessments.

**Results:**

At an average follow-up of 12.5 months, 88% of patients achieved postoperative symmetry, with preoperative asymmetry improving from 4.5 mm ± 1.9 mm to 0.8 mm ± 0.9 mm (*p* < 0.001). Medial asymmetry, brow peak asymmetry, and lateral brow asymmetry were also all significantly improved (*p* < 0.0001). Six patients (12%) had residual asymmetry >2 mm (mean: 2.5mm), but all had significant improvement from baseline. Complications included temporary frontalis apraxia (8%, resolved by 8 months) and sensory deficits (4%, resolved by 12 months), with no cases of permanent nerve injury or alopecia.

**Conclusion:**

A patient-specific approach in endoscopic brow lift with asymmetric ligament release and selective fixation yields better results for patients with asymmetric ptosis compared to conventional techniques. Understanding individual anatomical variations can help surgeons minimize the risk of overcorrection and achieve more natural-looking outcomes.

**Level of Evidence IV:**

This journal requires that authors assign a level of evidence to each article. For a full description of these Evidence-Based Medicine ratings, please refer to the Table of Contents or the online Instructions to Authors www.springer.com/00266.

**Supplementary Information:**

The online version contains supplementary material available at 10.1007/s00266-026-05718-1.

## Introduction

Asymmetric brow position is both an aesthetic and functional challenge, disrupting facial harmony and patients’ self-perception and satisfaction. The underlying causes of brow asymmetry are diverse, encompassing congenital structural variations, aging-related soft tissue laxity, facial nerve dysfunction, and compensatory adaptations to underlying eyelid ptosis [1]. Even subtle differences in brow height or contour can create unintended expressions of fatigue, anger, or sadness and prompt patients to seek surgical correction. However, achieving long-term symmetry is complex due to the dynamic interplay of brow elevator and depressor muscles and anatomic variations [2].

Endoscopic brow lift has emerged as a leading technique for addressing brow ptosis, offering a minimally invasive approach with favorable cosmetic outcomes and reduced morbidity compared to traditional open procedures [3]. Conventional brow lifting techniques often employ bilateral symmetric release with the assumption of uniform anatomical response to elevation. However, this standardized approach fails to account for preexisting facial asymmetry and frequently results in postoperative imbalances, including residual asymmetry, overcorrection of the less ptotic brow, or undercorrection of the more affected side [4, 5]. Given these challenges, a patient-specific approach that selectively addresses asymmetry through differential ligament release and targeted fixation may offer superior outcomes.

In this case series, we evaluate the efficacy of asymmetric ligament release and selective fixation in endoscopic brow lifting to address asymmetric brow ptosis. By tailoring the surgical approach to each patient’s unique anatomical characteristics, this method aims to optimize symmetry while minimizing the risks associated with traditional techniques. While experienced surgeons may titrate ligament release based on asymmetry [6], most published techniques still emphasize symmetric, bilateral, and total release [2, 4, 5, 7–10]. To our knowledge, no prior case series has systematically quantified or reported outcomes based on deliberate asymmetric dissection and fixation.

## Methods

### Study Design and Patient Selection

This retrospective case series analyzed consecutive patients with asymmetric brow ptosis who underwent endoscopic brow lifting performed by a single surgeon (S.R.) between January 2022 and December 2024. Patients were noted to have brow ptosis if the brow descended below the supraorbital rim or if the tail of the brow was lower than the medial brow at rest. Brow height was measured at 3 points using ImageJ digital analysis (NIH, Bethesda, MD): the medial-most point of the brow, the lateral-most point of the brow, and the peak of the brow. The peak of the brow was defined as either the highest point of the brow arch or if the brow was flat or downturned—the height of the brow at a point in the middle of the lateral limbus and the lateral canthus. Inclusion criteria required patients to have ≥ 2 mm of asymmetry between the peak of the brows. Patients with previous brow or upper eyelid surgery, facial trauma, eyebrow paralysis, or underlying disorders affecting brow positioning (e.g., asymmetric blepharoptosis (difference in MRD1 of ≥ 1 mm between eyelids), Bell’s palsy, myasthenia gravis) were excluded to minimize confounding factors. Preoperative assessment also included margin reflex distance 1 (MRD1) to evaluate for concurrent eyelid ptosis or dermatochalasis, as well as dynamic frontalis testing both at rest and during maximal voluntary contraction to evaluate for asymmetry or weakness. Cases were also reviewed for signs of Hering’s dependence or other compensatory mechanisms with the phenylephrine test in cases of significant unilateral eyelid ptosis.

The primary outcome of this study was postoperative brow symmetry, defined as a final asymmetry of < 2 mm between the peak of the brows at the final follow-up visit. Additional measures included medial and lateral brow symmetry, the incidence of overcorrection, or undercorrection, defined as persistent brow asymmetry exceeding 2 mm at any postoperative time point, as well as the rate of surgical complications, including temporary or permanent nerve dysfunction, wound healing complications, infection, alopecia, or frontalis muscle paresis.

All patients provided informed consent for the procedure, including the use of identifiable clinical photographs for research purposes. The study protocol adhered to the Helsinki Declaration on ethical principles for medical research and qualified for an IRB waiver.

### Surgical Technique

All procedures were performed under general anesthesia with patients in the supine position. A standardized endoscopic approach was used to minimize visible scarring and optimize visualization of the brow ligaments and periosteal attachments. Three surgical interventions were devised to address brow asymmetry.

**Fig. 1:**
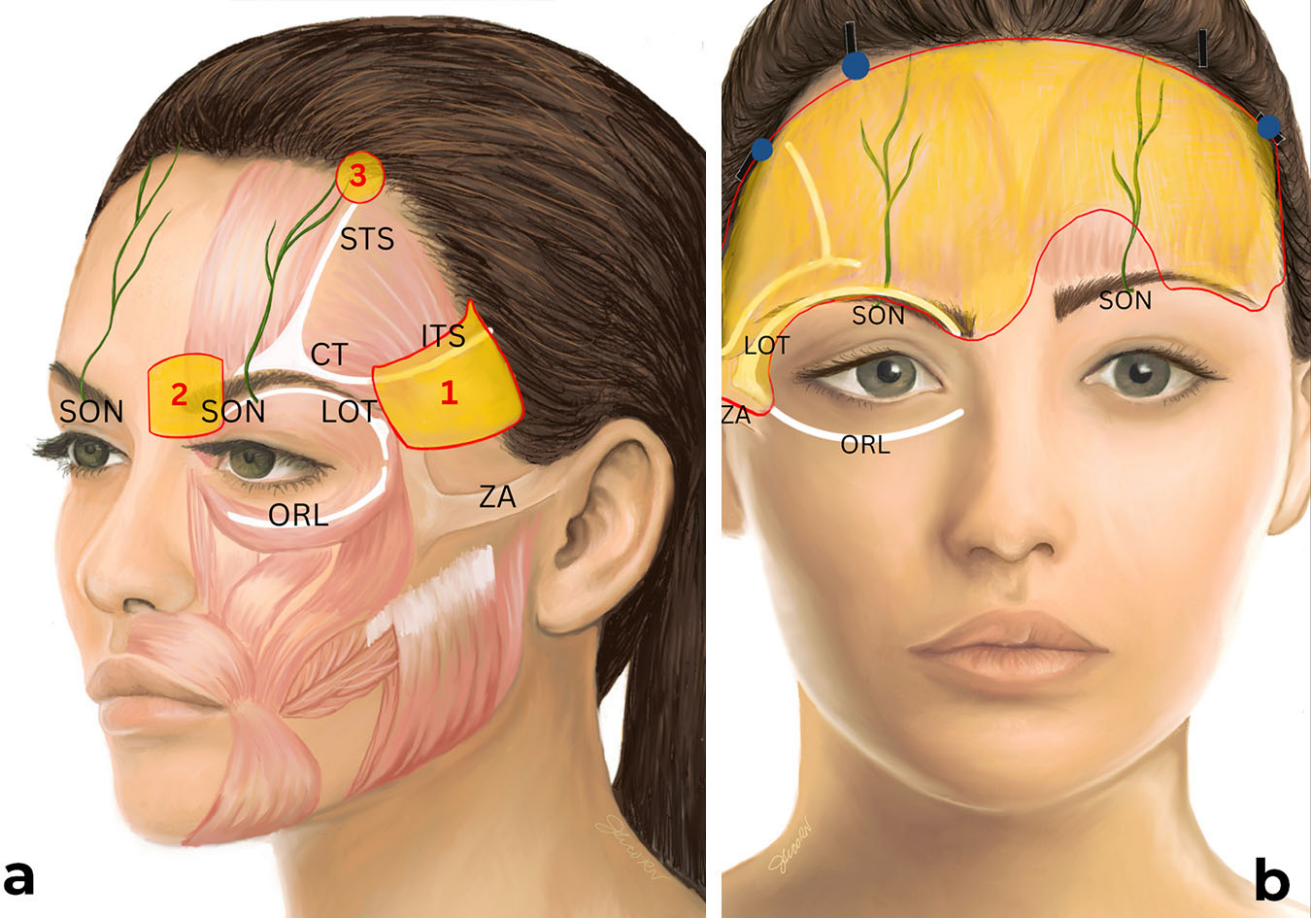
Schematic depiction noting (**a**) the three interventions: (1) extended lateral release, (2) release medially past the supraorbital notch, and (3) unilateral paramedian fixation, and showing (**b**) the typical release pattern in severe asymmetric brow ptosis. The release pattern on the right forehead is consistent with extended/maximal dissection, and the release pattern on the left is consistent with minimal/standard dissection. *SON* supraorbital nerve, *CT* conjoint tendon, *STS* superior temporal septum, *ITS* inferior temporal septum, *ZA* zygomatic arch, *ORL* orbital retaining ligament, *LOT* lateral orbital thickening.

Tumescent anesthetic solution was used to infiltrate the forehead. The surgical technique consisted of the following steps (Fig. 1).

*Incision Placement:* Four 1.5-cm scalp incisions (two temporal (sagittal) and two paramedian incisions (radial) on each side) were made posterior to the hairline to minimize visible scarring. The incisions were strategically placed to preserve vascular and neural structures. The paramedian incision specifically was placed at the desired high point of the brow.

*Subperiosteal Dissection:* A subperiosteal pocket was created over the frontal bone to the superior orbital rim and arcus marginalis, ensuring preservation of the supraorbital and supratrochlear neurovascular bundles.

*Asymmetric Ligamentous Release:* A dissection was performed over the deep temporal fascia over the temporalis muscle. The temporal fusion line was then released across the entire border of the temporalis muscle and temporoparietal bones. The lateral orbital thickening was also released to allow elevation of the tail of the brow. This standard temporal release was performed bilaterally in all patients, regardless of the degree of asymmetry for two reasons: (1) to address any latent lateral brow ptosis present and (2) to maintain symmetric frontalis muscle dynamics, where unilateral elevation may result in unnatural or asymmetric dynamic rhytids.

More elevation of the arcus marginalis was performed on the more ptotic side, and the release tapered toward the medial brow to create more lateral lifting power. On the more ptotic side only, dissection was carried laterally to the root of the helix to fully release the lateral retinaculum inferior temporal septa (Intervention 1) [6]. Care was taken to perform this release under endoscopic visualization to avoid trauma to the frontal branch of the facial nerve which is in a danger zone in this region.

Medially, the arcus marginalis was released to the supraorbital notch. On the more ptotic side only, release of the arcus marginalis was extended medially past the notch into the glabella and nasal dorsum, without releasing the tissues around the contralateral supraorbital bundle (Intervention 2). The medial brow musculature was not resected or disturbed surgically.

*Fixation Techniques:* The paramedian incisions were fixated to the calvarium of the frontal bone bilaterally in most cases, with a 4-0 Monocryl secured to bone tunnels. In cases of severe brow asymmetry as determined by the senior author, unilateral paramedian fixation was performed on the more ptotic side only (Intervention 3). These incisions were then closed with staples.

### Statistical Analysis

Descriptive statistics were used to summarize demographic data, preoperative asymmetry, and postoperative outcomes. Continuous variables (e.g., asymmetry measurements) were reported as means ± standard deviations, while categorical variables (e.g., complication rates) were expressed as percentages. Paired *t* tests were used to compare preoperative and postoperative asymmetry. Statistical significance was set at *p* < 0.05.

## Results

A total of 50 consecutive patients met the inclusion criteria. The mean patient age was 62.1 years (range: 30–74 years) with mean follow-up of 12.5 months. 98% of patients were female, consistent with the demographic seeking brow rejuvenation procedures in the author’s practice. Surgical interventions included extended lateral ligament release (lateral release to the helical root performed on 100% of ptotic sides), extended medial dissection (performed on 62% of ptotic sides), and unilateral paramedian fixation (performed on 25% of cases). Concomitant procedures performed included upper blepharoplasty (90%), lower blepharoplasty (80%), and face/neck lifting (48%).

**Fig. 2:**
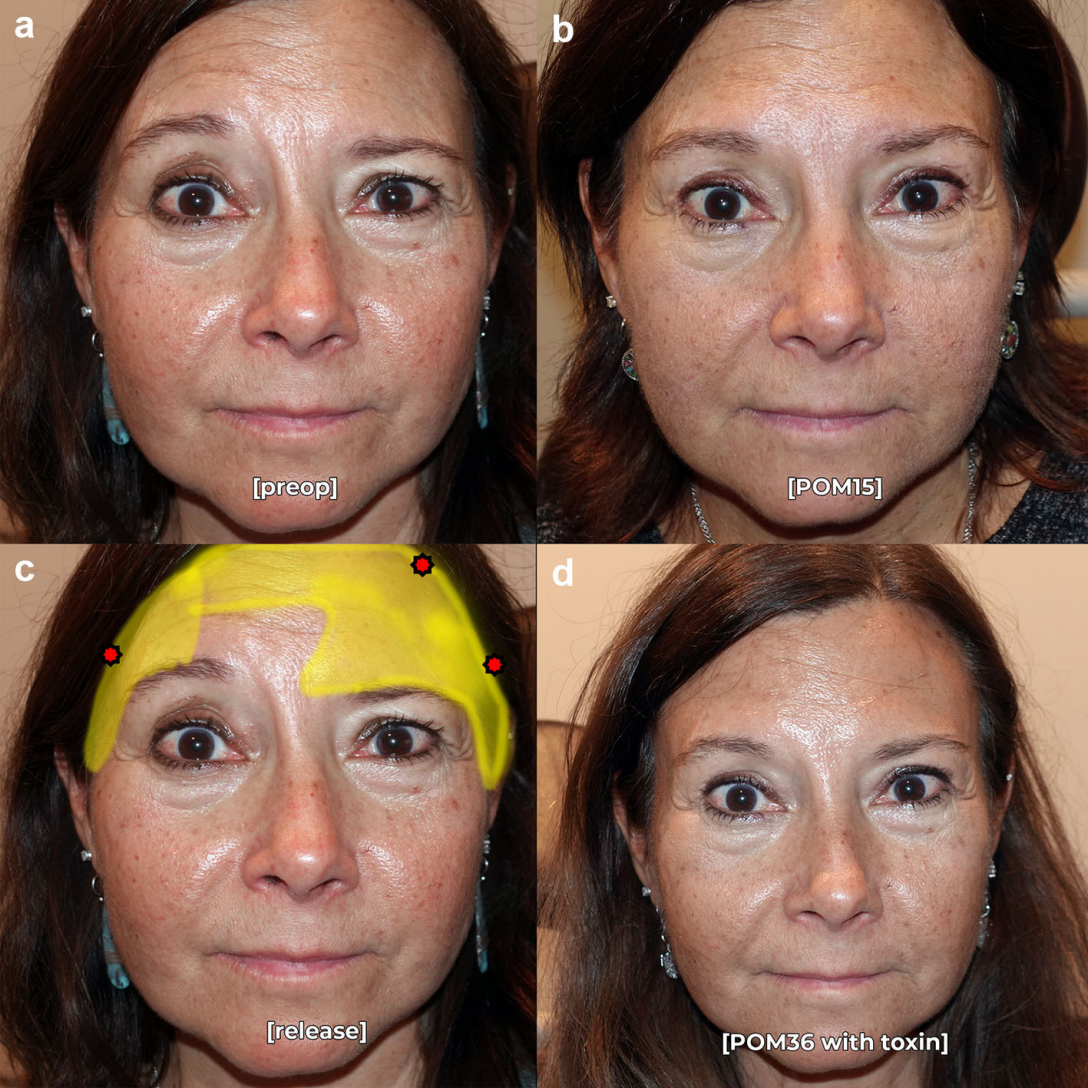
Pre- (**a**) and postoperative month 15 (**b**) photographs in repose of a 54-year-old female who underwent successful endoscopic brow recontouring with Interventions 1-3 (extensive lateral release, added medial release, and unilateral fixation). Release (yellow) and fixation points (red) are noted in **c**. Specifically, this patient’s brow asymmetry preoperatively was not responsive to botulinum toxin; the preoperative photograph shown is with her coach to not make any facial expressions. Photograph at postoperative month 36 (**d**) with botulin toxin on board shows improvement in dynamic frontalis rhytids, but no meaningful improvement in brow symmetry.

**Fig. 3:**
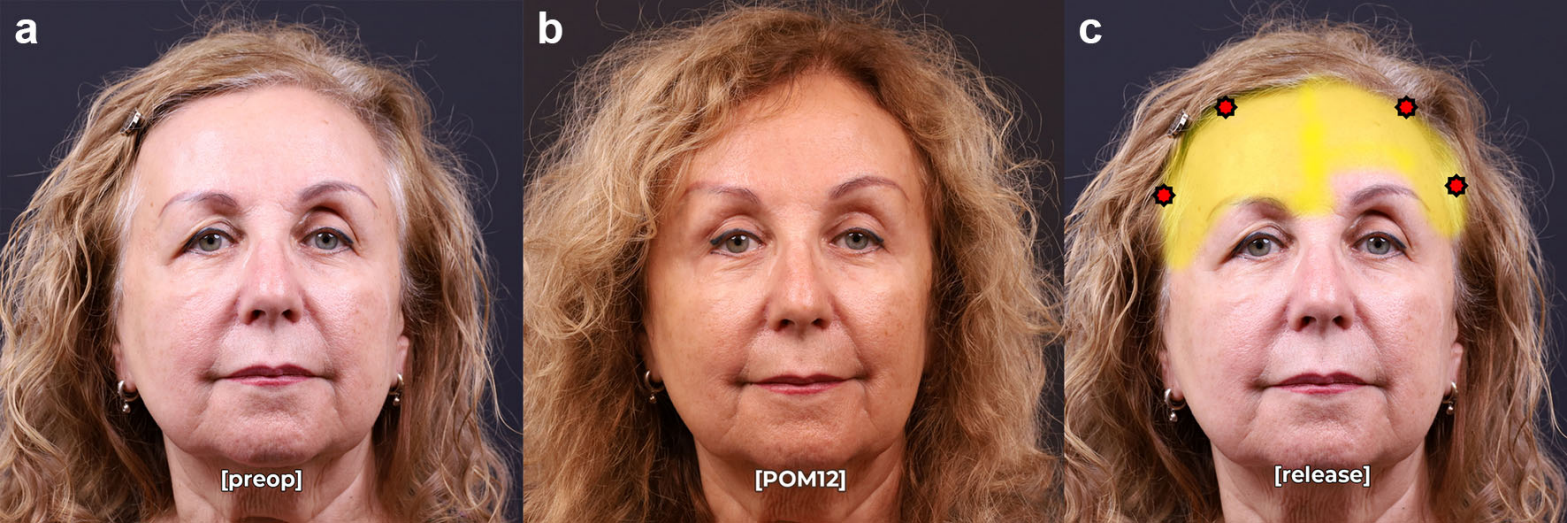
Pre- (**a**) and postoperative month 12 (**b**) photographs in repose of a 62-year-old female who underwent successful endoscopic brow recontouring, upper blepharoplasty, and superior sulcus fat grafting with Interventions 1-2 (extensive lateral release, added medial release) with concomitant upper blepharoplasty and superior sulcus fat grafting. Release (yellow) and fixation points (red) are noted in **c**.

**Fig. 4:**
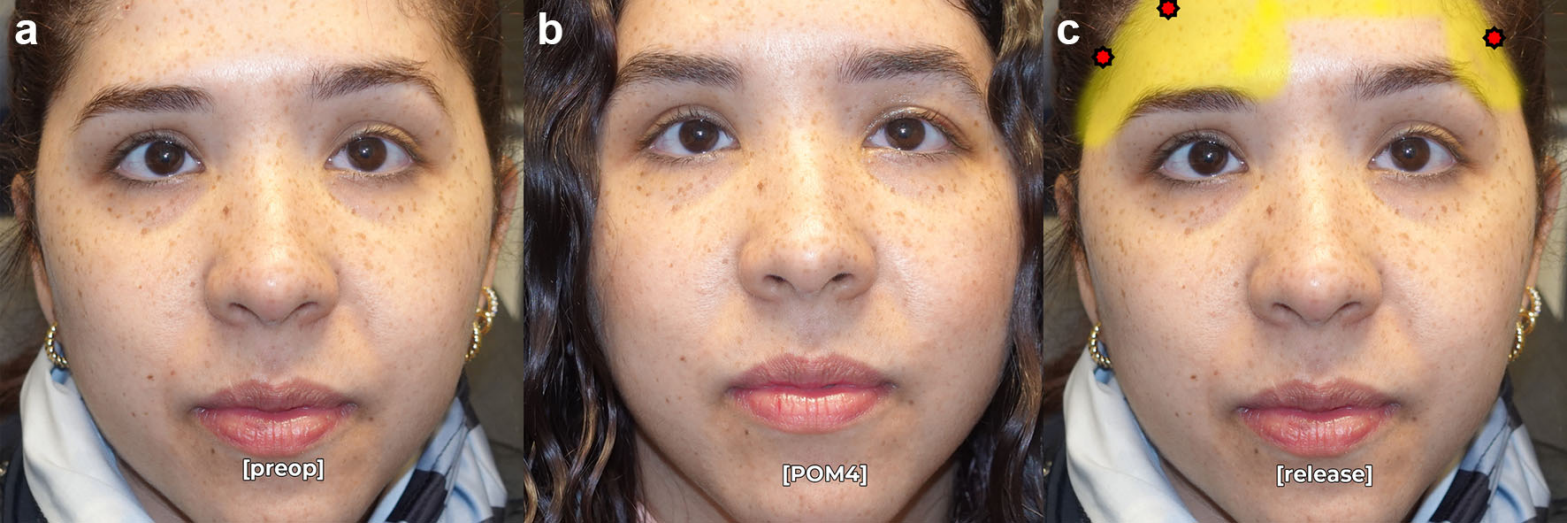
Pre- (**a**) and postoperative month 12 (**b**) photographs in repose of a 33-year-old female who underwent successful endoscopic brow recontouring with Interventions 1-3 (extensive lateral release, added medial release, and unilateral fixation). Release (yellow) and fixation points (red) are noted in **c**.

**Fig. 5:**
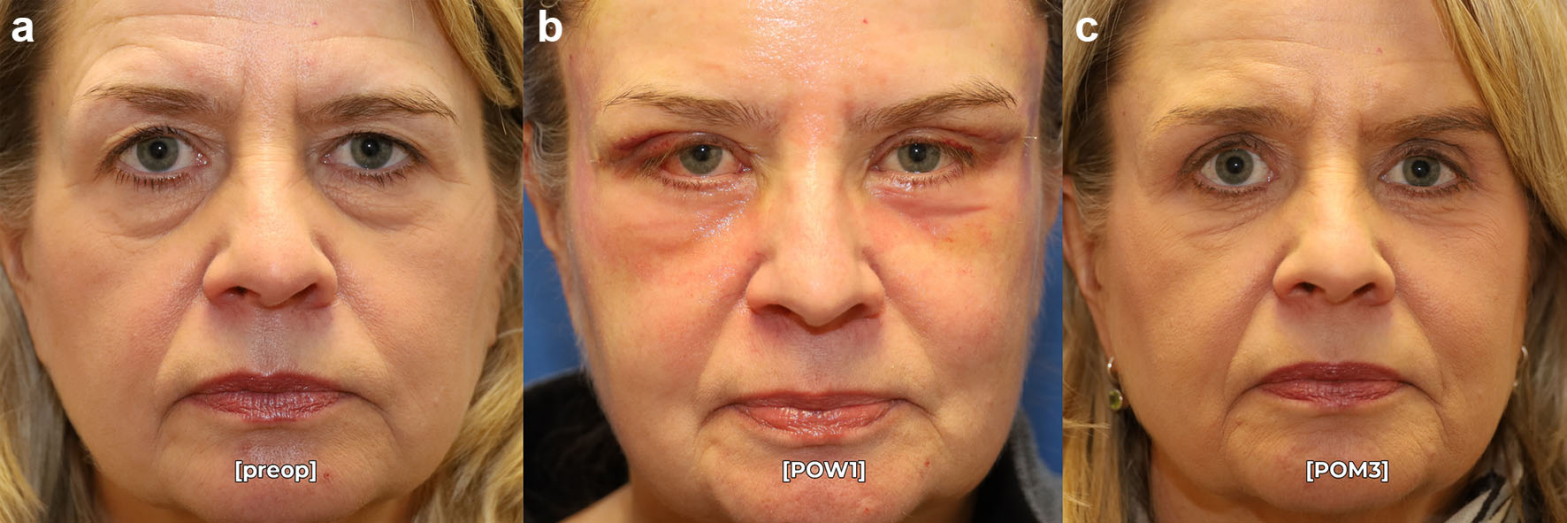
Pre- (**a**) and postoperative month 3 (**c**) photographs in repose of a 65-year-old female who underwent endoscopic brow recontouring, upper/lower blepharoplasty, and midfacial fat grafting with residual postoperative asymmetry. Of note, she has symmetry at postoperative week 1 (**b**), but has a hyperdynamic right frontalis muscle at postoperative month three causing residual brow asymmetry. This patient was classified as a failure in this surgical series.

**Fig. 6:**
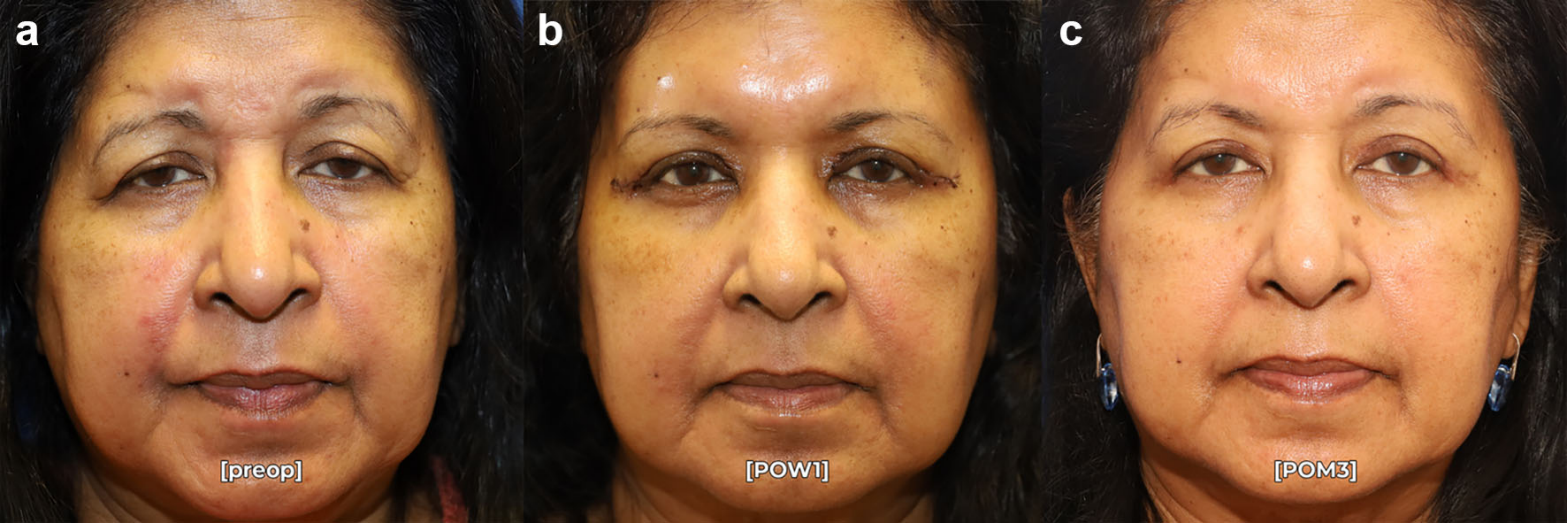
Pre- (**a**) and postoperative month 3 (**c**) photographs in repose of a 72-year-old female who underwent endoscopic brow recontouring and posterior-approach ptosis repair with residual postoperative asymmetry. Of note, she has symmetry at postoperative week 1 (**b**), but has a hyperdynamic left frontalis muscle and a shallow right superior orbital rim potentially causing residual brow asymmetry. This patient was classified as a failure in this surgical series.

Postoperative analysis demonstrated a significant improvement in brow symmetry among patients who underwent asymmetric ligament release and selective fixation. At an average follow-up of 12.2 months, 88% of patients achieved postoperative symmetry, defined as <2mm of asymmetry at the peak brow position. The mean preoperative brow peak asymmetry was 4.5 mm ± 1.9 mm, which improved to 0.8 mm ± 0.9 mm postoperatively (*p* < 0.001, Table 1). Medial brow asymmetry improved from 2.2 mm ± 1.6mm to 0.2 mm ± 0.8mm, and lateral brow asymmetry improved from 3.2 mm ± 1.6 mm to 0.4 mm ± 0.7 mm. All patients experienced significantly improved asymmetry compared to preoperative baseline (Figs. 2, 3, 4). Six patients had residual brow peak asymmetry ≥ 2mm, despite having symmetric brows intraoperatively and at the first postoperative visit (representative examples: Figs. 5, 6).

**Table 1:** Descriptive statistics of the three interventions and outcomes.

Intervention	%
Extensive lateral release	100
Extensive medial release	62
Unilateral fixation	25

Brow asymmetry	Preop (mm)	Postop (mm)	*p*
Medial brow asymmetry	2.2 ± 1.6mm	0.2 ± 0.8mm	<0.0001
Brow peak asymmetry	4.5 ± 1.9mm	0.8 ± 0.9mm	<0.0001
Lateral brow asymmetry	3.2 ± 1.6mm	0.4 ± 0.7mm	<0.0001

Complications	%
Asymmetry >2mm	n=6 (12%)
Prolonged apraxia (mean 8 months to resolution)	n=4 (8%)
Prolonged paresthesia (12 months to resolution)	n=1 (2%)

No major complications, such as wound dehiscence, alopecia, infection, hematoma, or permanent nerve injury, were observed. Minor complications occurred in a small subset of patients and resolved without long-term sequelae. Transient frontalis paresis was observed in 8% of patients (n = 4), characterized by mild weakness in brow elevation on the more ptotic side, which is completely resolved by 8 months postoperatively. Prolonged sensory deficit in the supraorbital or supratrochlear nerve distribution occurred in 4% of patients (n = 1), with gradual resolution by 12 months.

## Discussion

Management of the asymmetric brow remains a complex challenge in facial plastic and oculoplastic surgery, requiring a balance between aesthetic symmetry, functional restoration, and long-term stability. This case series highlights the efficacy of asymmetric ligamentous release and selective fixation in endoscopic brow lifting, offering an alternative to traditional release techniques that fail to account for individual anatomical variations. By adopting a tailored approach, this study provides evidence that patient-specific interventions yield more predictable and natural-looking outcomes, reducing the risk of overcorrection and residual asymmetry.

We aim to improve upon the traditional approach to endoscopic brow lifting [4] and show that a patient-specific, titrated ligamentous release is better for patients with asymmetric ptosis. A uniform full deep-plane release in all brow lift patients was the prior dogma, but such an indiscriminate approach can lead to overcorrection in some cases. Abraham et al. [11] reported a rate of 15-20% of postoperative brow asymmetry after uniform ligament release, which supports the idea that standardized surgical techniques may not be effective for individuals with diverse anatomical features. The key aspect of our method is the selective release of the retaining ligaments of the forehead, based on Knize’s [12] anatomical study that identified this retinacular network as the main structure preventing upward movement of the brow.

Our results support the effectiveness of asymmetric ligament release in achieving improved postoperative symmetry, with 88% of patients attaining postoperative brow symmetry (asymmetry ≤2mm). This represents a significant reduction from the mean preoperative asymmetry of 4.6 mm, reinforcing the idea that targeted ligament lateral release and thoughtful fixation provide superior control over brow height and contour. The selective additional release of medial ligaments (62% of cases) demonstrated that medial dissection is not always necessary and should be reserved for cases of medial brow ptosis, thereby avoiding the “surprised” look that can result from excessive medial brow elevation [3, 13]. Moreover, the medial ligamentous release alone appears to be sufficient to address medial brow position in this cohort, without traumatizing the corrugators and procerus which can risk splaying the brows, causing paresthesia, or contour deformities [14]. Similarly, unilateral paramedian fixation (25% of cases) proved effective in refining asymmetry without destabilizing the contralateral brow. While fixation was sometimes asymmetric, bilateral dissection and temporal fixation were performed in all patients. This strategy aligns with previous biomechanical studies, which suggest that asymmetrical suspension allows for finer titration of brow elevation while preserving natural muscle dynamics [13]. In our experience, bilateral intervention minimizes the risk of asynchronous frontalis activity and may prevent unveiling of latent brow ptosis in older patients.

While our technique did not incorporate vertical midline periosteal division, this maneuver may offer additional mobility in select cases. We intentionally preserved the midline periosteum to maintain central brow stability and avoid unintended distortion of glabellar contours. However, in patients with severe asymmetry or inadequate response to lateral release and unilateral fixation, midline division could theoretically allow for additional titration of lift across the paired frontalis muscle bellies.

Our study reinforces the safety of a patient-specific approach to brow lifting, with no major complications such as permanent nerve injury, wound dehiscence, or infection. Minor complications were transient and self-limiting, including temporary frontalis paresis (8%) and prolonged sensory deficit (4%), both of which resolved within 8–12 months, consistent with the neuropraxia model of nerve injury [15, 16]. Notably, our approach to conservative medial ligament release may have contributed to a lower rate of sensory disturbances compared to studies reporting 5–10% rates of supraorbital neuropathy following aggressive dissection near the supraorbital notch [17]. Notably, this modification does not protect against injury to the deeper supraorbital nerve branch adjacent to the conjoint tendon. [12]

Six patients in our cohort had residual brow asymmetry, although their symmetry was still improved from the preoperative state. Interestingly, this was not a failure of technique as the patients were noted to be symmetric both intraoperatively, and at postoperative week 1 when the brows were still apraxic (Figs. 5, 6). This suggests that dynamic factors such as frontalis muscle hyperactivity and variable insertion of the muscle across the hemifaces may account for residual postoperative brow asymmetry. Further asymmetry may be caused by underlying bony changes that provide inadequate support to the more ptotic brow. These patients may be successfully treated with adjunctive botulinum toxin [18]. However, it is important to note that no patients had worsening asymmetry after the asymmetric brow lift technique described in this manuscript. Even patients with significant dynamic brow asymmetry (Figs. 2, 5) can be *improved* with this technique and allows for a better aesthetic result in conjunction with postoperative toxin treatment.

Despite its promising findings, this study has several limitations. Firstly, as a retrospective case series, it is inherently subject to selection bias, limiting the ability to establish causality or direct comparisons with traditional deep-plane release techniques. A prospective, randomized controlled trial would be necessary to validate these findings and determine whether asymmetric ligament release provides superior long-term outcomes.

Secondly, our predominantly female cohort restricts generalizability to male patients, who have thicker forehead musculature, denser retaining ligaments, and different aesthetic goals. Abraham et al. [11] reported that male patients needed 30% wider dissection to achieve similar brow lift, indicating that surgical protocols may need to be sex-specific. Future studies should assess whether sex-based anatomical differences necessitate modifications to ligament release or fixation techniques.

Thirdly, our study relied on 2D digital photographic analysis for symmetry assessment, which, while widely used, carries inherent limitations. While all efforts were made to capture pre- and postoperative photographs with patients in a relaxed, neutral expression, minor differences in frontalis activation may confound visual assessment of brow position in some cases. Recent studies suggest that 3D photogrammetry can reduce asymmetry measurement error by up to 0.8 mm, providing more reliable postoperative assessments [19]. Incorporating advanced imaging modalities and even videos to understand dynamic brow position in future research could refine surgical planning and improve outcome analysis.

Fourthly, concomitant upper eyelid surgery may have altered factors that affect dynamic brow position. The decision to include patients undergoing concomitant upper eyelid surgery was made as most patients with brow ptosis also typically benefit from upper eyelid surgery. Finally, the link between ptosis and brow position is not clear, and frontalis muscle activation may occur both ipsilateral and contralateral to the more ptotic eyelid. [20] However, to minimize any potential interactions, patients with significantly asymmetric blepharoptosis were excluded from this study.

The average follow-up of 12.5 months in this study precludes from making conclusions about the long-term durability of asymmetric brow correction. While Monocryl suture has a shorter half-life than comparable monofilament absorbable sutures such as PDS, we did not note any loss of fixation at the wounds in this cohort during the follow-up period. Lee et al. [21] reported a 15% relapse rate in endoscopic brow lifts at two years, mainly due to gradual ligament reattachment and changes in the frontalis muscle compensation. Long-term follow-up is necessary to determine whether the hypothesized asymmetric release technique provides long-term correction or secondary interventions become necessary over time.

## Conclusion

In conclusion, this study demonstrates that asymmetric ligament release and selective fixation in endoscopic brow lifting provide a safe, effective, and patient-specific approach for treating asymmetric brow ptosis. By tailoring surgical techniques to individual anatomical variations, surgeons can reduce overcorrection, minimize residual asymmetry, and enhance aesthetic outcomes. Our results highlight the value of a precision-driven approach over traditional bilateral release, which often overlooks the complex interplay of facial anatomy. Future research should focus on enhanced preoperative planning tools, such as 3D imaging and muscle activity analysis, as well as long-term follow-up to assess the durability and adaptability of this technique over time.

## Supplementary Information

Below is the link to the electronic supplementary material.

Supplementary file1 (MP4 423502 kb)
